# Prospects for direct social perception: a multi-theoretical integration to further the science of social cognition

**DOI:** 10.3389/fnhum.2014.01007

**Published:** 2015-01-07

**Authors:** Travis J. Wiltshire, Emilio J. C. Lobato, Daniel S. McConnell, Stephen M. Fiore

**Affiliations:** ^1^Cognitive Sciences Laboratory, Institute for Simulation and Training, University of Central FloridaOrlando, FL, USA; ^2^Department of Psychology, University of Central Florida, OrlandoFL, USA; ^3^Department of Philosophy, University of Central FloridaOrlando, FL, USA

**Keywords:** social cognition, direct perception, social affordances, embodied neuroscience

## Abstract

In this paper we suggest that differing approaches to the science of social cognition mirror the arguments between radical embodied and traditional approaches to cognition. We contrast the use in social cognition of theoretical inference and mental simulation mechanisms with approaches emphasizing a direct perception of others’ mental states. We build from a recent integrative framework unifying these divergent perspectives through the use of dual-process theory and supporting social neuroscience research. Our elaboration considers two complementary notions of direct perception: one primarily stemming from ecological psychology and the other from enactive cognition theory. We use this as the foundation from which to offer an account of the informational basis for social information and assert a set of research propositions to further the science of social cognition. In doing so, we point out how perception of the minds of others can be supported in some cases by lawful information, supporting direct perception of social affordances and perhaps, mental states, and in other cases by cues that support indirect perceptual inference. Our goal is to extend accounts of social cognition by integrating advances across disciplines to provide a multi-level and multi-theoretic description that can advance this field and offer a means through which to reconcile radical embodied and traditional approaches to cognitive neuroscience.

## Introduction

Although the embodied approach to cognition has made great strides shifting the consensus of how perception, action, and cognition are viewed across a number of disciplines, the role of mental representations in such processes is still highly debated (e.g., Chemero, 2009; Wilson and Golonka, 2013). Such divergence in perspectives may suggest that embodied cognitive neuroscience requires an integrative account that allows room for both representational and non-representational perspectives (cf. Dale, 2010; Horton et al., 2012). A key point of contention between these perspectives centers on whether or not perception can be direct or must necessarily be indirect (cf. Ullman, 1980). Similar arguments have been made in the field of social cognition (see Barrett et al., 2007; Bohl and van den Bos, 2012). In particular, this field is typically united through its focus on the perceptions, actions, and cognitive processes involved during observations and interactions between conspecifics (Frith and Frith, 2007); however, because of the large number of disciplines and perspectives involved (e.g., social and cognitive psychology, neuroscience, and philosophy), the mechanisms underlying social cognitive processes remain highly debated.

The specific debate often centers on the ways in which humans are able to understand the mental states of others, whether through inferential mechanisms (e.g., Gopnik and Wellman, 1992), simulation mechanisms (e.g., Blakemore and Decety, 2001; Goldman, 2006), and/or direct perception (Gallagher, 2008; De Jaegher, 2009). This is the core of the present article: to provide an integrated theoretical account of social cognitive mechanisms, with a specific focus on the role of direct perception. Recent integrative accounts suggest that humans have the capability to employ each of these mechanisms, though, in differing contexts (e.g., Beer and Ochsner, 2006; Bohl and van den Bos, 2012). This has been argued through theorizing that relies on findings from social cognitive neuroscience (Bohl and van den Bos, 2012), by incorporating the findings of two generally distinct types of cognitive processes useful for characterizing social cognition (Satpute and Lieberman, 2006; Lieberman, 2007; Adolphs, 2009). We suggest that this account of social cognition is limited in the extent to which it fully elaborates on the prospects for and limitations of direct social perception.

To address this limitation, we draw from a complementary set of theories that have not yet been fully integrated. As such, our aim is to extend accounts of social cognition by leveraging advances across disciplines to provide a multi-level and multi-theoretic description of social cognition that can also be informative to the integration of conflicting views in embodied cognitive neuroscience. This will, necessarily, mean that the following paper will cover theoretical breadth rather than depth as we acknowledge that multiple approaches are needed in order to attempt a more holistic understanding of social cognition. Table 1 provides an overview of the scope of the theoretical approaches addressed in this manuscript in accord with Repko’s (2008) guidelines for theoretical integration. Specifically, Table 1 is included here to detail the major theories that contribute to the overarching framework, key assumptions from each viewpoint, level of the framework where each theory provides insights, and the associated underpinning mechanisms of social cognition. Each of these is elaborated on throughout in more detail. We assert that the integration of these perspectives will ultimately lead researchers to consider a more holistic approach to the science of social cognition and further the contributions of the radical embodied approach.

**Table 1:** **Theoretical perspectives relevant to integrative account of social cognition**.

Theory	Key assumptions	Level(s) of framework	Underpinning mechanisms	Authoritative references
**Theory of mind**	• Extra-perceptual mechanisms are required for understanding another’s mental states			• Baron-Cohen (1995) • Gopnik and Wellman (1992)
	• Third-person mindreading— observational rather than interactive form of understanding others	• Sub-personal • Personal	• Inferential simulation • Theoretical inference	• Blakemore and Decety (2001) • Goldman (2006) • Frith and Frith (2007, 2008, 2012)
	• Individualistic cognitive processing			• Saxe et al. (2004)
**Interactionism**	• Mental states are directly perceivable through a person’s embodiment			• De Jaegher (2009) • De Jaegher et al. (2010)
	• Social cognition at the supra-individual as evolving between interactions	• Sub-personal	• Embodied intentionality
		• Personal • Supra-individual	• Social affordances	• Gallagher (2008) • Gangopadhyay and Schilbach (2012)
	• Cognition and perception are for actively relating to the environment			
**Dual process theories**	• Two distinct types of cognitive processes		• Type 1 cognitive processes— automatic and stimulus driven	• Evans (2008)
			
	• Type 1 processes are evolutionarily older	• Sub-personal • Personal	• Type 2 cognitive processes—controlled and flexible	• Frith and Frith (2007) • Lieberman (2007)
	• Type 2 processes are evolutionarily recent
**Ecological psychology**	• Body and environment play a constitutive role in understanding the social world		• Direct perception via dorsal visual system	• Chemero (2003) • Gibson (1979)
	• Perception is not inferential	• Sub-personal	• Kinematic Specific Dynamics	• McArthur and Baron (1983) • Norman (2002)
	• Perception and cognition serve an adaptive function providing an organism with means for direct interaction with the environment	• Personal • Supra-individual	• Perception-action loops	• Runeson and Frykholm (1983) • Valenti and Good (1991)
**Enactive cognition**	• Perceptions are actively brought forth through engagement with the environment
	• Perception is an active sense-making process that prepares an organism for action	• Sub-personal • Personal • Supra-individual	• Participatory sense-making • Social interaction	• De Jaegher and Di Paolo (2013) • Froese and Ziemke (2009) • Noë (2004)
	• Perception of invariant information relies on specific motor actions			
**Brunswik’s lens model**	• Proximal stimuli are perceivable features of the environment	• Sub-personal	• Probabilistic functionalism	• Brunswik (1956) • Cooksey (1996)
	• Distal features are objective states of the environment, not necessarily perceivable	• Personal • Supra-individual	• Causal ambiguity	• Doherty and Kurz (1996) • Vicente (2003)
**Dynamical systems**	• Interactions follow dynamic laws that structure and constrain joint perception-action systems in self-organizing patterns	• Sub-personal • Personal • Supra-individual	• Coupling of perception- action systems • Coupling of organism to environment	• Marsh et al. (2009a) • Marsh et al. (2006, 2009b) • Vallacher and Nowak (1997)
	• Dynamic laws are emergent across all size ranges of social units over varying temporal scales			• Richardson et al. (2014)

First, we provide an overview of the various disciplinary approaches to social cognition and then detail how these have been recently integrated into a coherent framework. We then express some of the limitations of that framework and describe how to address these. Namely, we argue that the perceptual basis for social cognition requires more attention with regard to prospects for what kind of social information can and cannot be directly perceived. Second, we define two complementary notions of direct perception: the embodied and embedded view of direct perception, which often focuses on social affordances (McArthur and Baron, 1983; Zebrowitz and Collins, 1997; Baron, 2007) and the enactive view of direct perception that argues for cases where mental states may be perceived directly (Gallagher, 2008; De Jaegher, 2009; Di Paolo and De Jaegher, 2012; Gangopadhyay and Schilbach, 2012). Third, we then elaborate on the informational basis for social perception by detailing the kinematic specification of dynamics (KSD) principle, a framework of social cues and social signals, situation semantics, the distinction between projectable and non-projectable properties, and the potential neural basis for direct social perception. Lastly, we attempt to integrate frameworks of direct and inferential perception from ecological psychology into the context of social perception and understanding of social interaction dynamics. We suggest that effective processing of social information is foundational to successfully interacting with others. We do not delve deeply into the variety of purposes of social cognition, writ large, such as managing relationships (e.g., Fiske, 1992; Beckes et al., 2014; Bohl, 2014), rather, we suggest that even basic and short-term interactions are contingent on the utilization of social information. Our goal is, not only to provide a more detailed articulation of some of the foundational mechanisms for social cognition, but also to advance a research agenda useful for integrating the radical embodied and traditional representational view of cognition. We next review long-standing and more recent accounts of social cognition.

## Disciplinary perspectives on social cognition

Since social cognition emerged as a theoretical and empirical area of research, the prevailing views incorporated traditional information processing perspectives on cognition. These approaches considered the brain as the mechanism through which complex mental representations of the social environment served the adaptive needs of an individual. Emerging from dominant views within computational and information processing theories of cognition, such approaches focused on the role and functions of the central nervous system when dealing with the social environment. Developmental, cognitive, and neuroscientific findings relevant to understanding social cognition have thus been interpreted in the context of the brain’s capacities for complex mental representations. As such, proposed mechanisms of social cognition have centered on how the brain represents the social world to facilitate understanding. The dominant mechanisms proposed have been folk psychological theoretical inference mechanisms (Gopnik and Wellman, 1992), and mental simulation mechanisms (Gallese and Goldman, 1998; Goldman, 2006). More recently, others have challenged this view to take into account the role of the body and interaction with others in the environment. To review these approaches, we first summarize Theory Theory (TT) and Simulation Theory (ST) accounts of social cognition. We then discuss more recent embodied accounts of social cognition and conclude with interactionist’s accounts of social cognition.

### Theory of mind—theory theory and simulation theory

Drawing from developmental psychology and neuroscience, two proposed mechanisms have been largely dominant in discussions of social cognition. These mechanisms focus on the roles of folk psychological theorizing and mental simulation to advance knowledge on how humans can understand the mind of another. Accordingly, the theoretical paradigms built up from these proposed mechanisms are known as TT (see Gopnik and Wellman, 1992, 2012) and ST (see Blakemore and Decety, 2001; Goldman, 2006). Whereas TT posits that we develop an inferential theory of others’ mental states to understand them, ST argues there is no need for such theories as one can use their own mind as a model to understand another’s mind (cf., Gallagher, 2008). Here, it should be noted that “simulation” as used by proponents of ST refers to a mental representation of another person’s behavior, and not to be confused with “embodied simulations,” which are non-introspective simulations that assume direct mapping of perception to action (see Gallese, 2007, 2009) or activations of motor representations (e.g., Barsalou, 2008). A fundamental assumption within both TT and ST approaches to social cognition is that the underlying mental states of another person must be worked out cognitively in order to understand them; the so-called “access problem” (Gangopadhyay and Miyahara, 2014). Specifically, both approaches describe processes of reasoning and judgment humans use to understand others’ mental states. In short, a mediating interpretational process—theorizing or simulation—is argued to be the means for understanding the intentions and mental states of others (see Di Paolo and De Jaegher, 2012).

Theory theory and ST approaches have provided rich, detailed accounts of social-cognitive processes, but there are two primary challenges to this area of theorizing—coming from both behavioral and neuroscientific work. On the behavioral side, one concern has to do with the nature of the interaction typically employed in traditional theory of mind (ToM) studies. On the neural side, a related concern has to do with the lack of specificity about the nature of the ostensive mentalizing network. We next briefly review these concerns.

At issue from the behavioral standpoint, many ToM studies have been criticized as being limited in their explanatory power because they adopt a “third person” observational perspective to social cognition (Di Paolo and De Jaegher, 2012; Przyrembel et al., 2012). That is, to a large degree, research adopting a TT or ST stance to social cognition requires that participants make observational social judgments in regards to stimuli, such as pictures or video clips, as opposed to an active engagement in social interaction. This approach to social cognition research elides the fact that social cognition primarily occurs during interactions, where a person is actively using their social-cognitive abilities to favorably advance the interaction.

Relatedly, recent research in neuroscience has shown that different brain regions are active in response to social “observation” compared to social “interaction” (Tylén et al., 2012). Using a combination of functional magnetic resonance imaging (fMRI) and eye-tracking, this research found that when participants observe a person manipulating an object, regions of the medial prefrontal cortex, the right inferior frontal gyrus, and the right inferior parietal lobule activate in response. In contrast, when viewing a character showing an object to the participant, meant to mimic a simple social interaction, activation in the right posterior temporal sulcus increases. Though, in both conditions, participants viewed video clips, the ostensive cues associated with showing an object to participants, compared to just manipulating the object, appear to have transformed the experience from a purely observational task to an interactive one (cf. Wilms et al., 2010). Further, when considering the fairly large body of work in neuroscience on ToM, the mentalizing network appears quite vast, involving a large number of regions and inter-connections. Indeed, according to a recent meta-analysis of neuroimaging studies on ToM (Mar, 2011), the mentalizing network includes the medial prefrontal cortex, the bilateral posterior superior temporal sulcus, bilateral angular gyri, bilateral anterior temporal areas, posterior cingulate cortex, precuneus, and the inferior frontal gyrus. However, as noted, an important qualifier here is that most neuroscience research interested in social interaction has only studied “pseudo interaction” (Przyrembel et al., 2012). That is, while research like that by Tylén et al. (2012) is a step in the right direction towards studying social interaction, it should be taken as tentative empirical evidence for neural regions associated with social cognitive processes given that the activations may be different during real social interaction.

There have even been attempts to synthesize TT and ST into hybrid accounts (e.g., Carruthers and Smith, 1996; Nichols and Stich, 2003), noting the possible roles of both mechanisms in social cognition. Though neuroscientific findings have been used to support TT, ST, and hybrid accounts, the evidence marshaled in favor of these accounts does not appear sufficient to favor one view over another. This suggests a need for social cognition researchers to establish new models of social cognition (see Apperly, 2008). We support this line of thinking to argue that these issues converge on a need for social cognitive neuroscience to move beyond the ST/TT accounts and provide a more comprehensive view of social cognition. While acknowledging the role of TT and ST mechanisms for understanding social cognition, assigning primacy to either or both of these mechanisms has been a significant criticism from opponents of these views (Di Paolo and De Jaegher, 2012). Instead, shifting the focus of social cognition research to include embodied forms of social interaction may be necessary in order to further advance the scientific understanding of social cognition (see Schilbach et al., 2013; Schilbach, 2014).

### (Embodied) social cognition

While the aforementioned work has contributed significantly to our understanding of social cognition, at issue is that this more cognitivist paradigm does not adequately consider the influence of the rest of the body, or the environment, on cognition. Conflicting theoretical accounts argue that the brain may not require complex amodal mental representations of the social world in order for the individual to understand it and engage in appropriate adaptive behaviors. This new paradigm emphasizes the importance of embodied and socially situated cognition (see Varela et al., 1991; Chemero, 2013; Semin and Smith, 2013; Wilson and Golonka, 2013).

In some cases, cognitivist accounts of cognition have been refashioned to better account for the role of the body and environment, by suggesting that, rather than abstract and amodal mental representations, such representations are inherently linked to their perceptual modality (e.g., Barsalou, 2008, 2010; Pezzulo et al., 2011) and these modal representations are what enables understanding of the social environment (Niedenthal et al., 2005). This form of grounded cognition (Barsalou, 2008, 2010) emphasizes the role of bodily states, situated actions, and the environment on cognition, though it still maintains that cognition results from the processing of mental representations, albeit linked to neural representations of modality.

In other cases, the embodied cognition paradigm posits, more radically, that both an individual’s body and environment are all that is needed for social cognition. They argue that there is no need for the brain to constantly maintain highly complex mental representations, whether modal or amodal, in order to understand the social world (Barrett et al., 2007; Marsh et al., 2009b). That is to say that the body and the environment play a constitutive role, and, thus, lighten the cognitive responsibilities of the brain (Wilson, 2002). In such accounts, the resources available to an individual include the brain, body, environment, and their relationships, underpinned by tightly coupled perceptual motor systems. More generally across these accounts, theories of embodied cognition posit that perception and action are deeply interrelated in such a way that the brain and body actively rely on, and make use of, the environment for effective interactions (e.g., Marsh et al., 2006; Barsalou, 2008).

Relevant to social cognition, this embodied approach has encouraged the development of theories that explain how the environment provides a person with the information necessary to directly perceive and understand socially relevant information, particularly during interaction (Gallagher, 2008; Gallagher and Varga, 2013). This theoretical position argues that social beings rely primarily on perceptual processes, and not necessarily on extra-perceptual, or more conceptual processes, in order to understand the social world (cf., Bowlby’s (1969) discussion of perceptual building bricks; see also IJzerman and Koole, 2011). Likewise, the embodied perspective has allowed for reinterpretations of developmental, cognitive, and neuroscientific findings that provide a more cohesive and cogent framework of social cognition, taking into consideration the role of the body and the environment, as well as the brain’s ability to perform complex operations to understand the social world (e.g., Bohl and van den Bos, 2012).

### Interaction theory

In contrast to TT and ST approaches, and more in line with embodied theories, *Interaction Theory* (IT) features social cognition during interaction more prominently (Gallagher, 2008). Additionally, unlike proponents of strong versions of TT and ST, IT theorists do not assume the unobservability of others’ minds^1^. That is, IT theorists posit that the mental states of others can be directly perceivable, without necessarily needing to call upon extra-perceptual processes, such as inferential theorizing or mental simulation (Gallagher, 2008; Gallagher and Varga, 2013). According to IT, the social information perceived during interactions allows for the immediate and rapid understanding of the beliefs, emotions, and intentions of other agents. Notably, the emphasis on social interaction has led some theorists to submit that the experience of direct perception of others’ mental states may only be possible during social interaction (e.g., De Jaegher, 2009).

IT-based views have emerged in social cognitive neuroscience that elaborate on the kind of information that support this direct perceptual grasp of another’s mental states. For example, Gangopadhyay and Schilbach (2012) posit that during social interactions, humans are able to directly perceive others’ mental states through perception of their *embodied intentionality*. In other words, during interaction, the body presents richly structured information conveying to a perceiver certain types of social interaction. This notion is similar to what we will later describe as *social affordances*. However, on this view, it is hypothesized that motor resonance mechanisms, defined as the “automatic activation of motor control systems during perceptions of actions” (Chaminade and Cheng, 2009, p. 287), underpin the direct perception of mental states and further present unique opportunities for action, interaction, and coordination (Gallagher, 2008; Gangopadhyay and Schilbach, 2012). Motor resonance mechanisms are thought to draw from the mirror neuron system; a system comprised of a special class of neurons which show activation during actions taken by an individual as well as the observation of the same action taken by another (Mukamel et al., 2010). Not only do such systems support mirroring behaviors, but also, motor resonance mechanisms are claimed to allow for a more direct grasp of others’ intentions (Iacoboni et al., 2005; Rizzolatti and Sinigaglia, 2010)^2^ and direct perception of affordances (Garbarini and Adenzato, 2004). According to IT, not only does embodied intentionality provide information regarding the mental states of others, but when the intentionality is shared, such as when working towards a common goal, the information constrains the temporal and coordinative dynamics of the interaction (Gangopadhyay and Schilbach, 2012), such as those required for joint action (Sebanz et al., 2006).

In sum, IT contributes to an integrated account of social cognition through its embodied view of cognition, its emphasis on the direct perception of others’ mental states and opportunities for interaction, its linkage to neural mechanisms, and lastly, by its emphasis on crucial factors of social interaction such as context and environment (Bohl and van den Bos, 2012). We turn now to a discussion of how the aforementioned theories can be more fully integrated in service of providing a more robust account of social cognition.

### Integrating, TT, ST, embodied and interactionist theories

**Figure 1:**
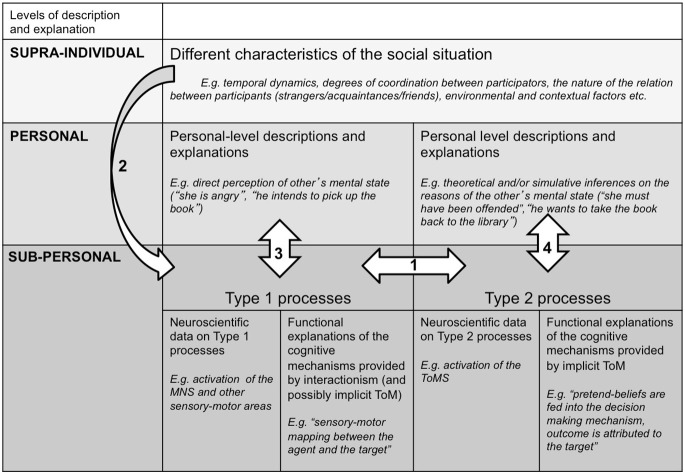
**Bohl and van den Bos’s 2012 integrative framework for social cognition (Figure as originally published in Bohl and van den Bos (2012))**.

Given the state of social cognition research, encompassing a large body of behavioral and neuroscience literature, researchers have recently attempted to integrate and synthesize these approaches into a coherent framework as a means of systematically exploring social cognition. One such framework has been posited by Bohl and van den Bos (2012), which utilizes a multi-layered framework that spans the sub-personal, personal, and supra-individual levels in order to understand the roles of theorizing, simulation, and direct perception in social cognition (see Figure 1). Personal processes are those at the level of the individual and her behavior, describable using intentional language. This level is most familiar to social cognition researchers, as the personal level is the most common target of social cognition research. Attributions of “She is sad” or “I want this paper to get published” are person-level attributions. Both TT and ST posit mechanisms that allow for mental state understanding at the personal level, as it is the individual who either theorizes about, or mentally simulates, the behavior of another agent. Sub-personal processes are those at the level of nervous system functioning, such as neuronal activation. This is the focus of social cognitive neuroscience research, typically. This distinction between the personal and sub-personal levels has a long history in the cognitive science literature (e.g., Dennett, 1969), upon which Bohl and van den Bos (2012) have extended to include the supra-individual level. The supra-individual level processes are those found outside the individual, such as environmental or situational contexts as well as temporal dynamics.

By incorporating dual-process theories of cognition into the overall framework, this multi-layered approach highlights crucial distinctions between those levels, providing data rich explanatory power to social cognition researchers. Likewise, this framework extends recent integrative accounts beyond the level of the individual (cf. Beer and Ochsner, 2006). Our aim here is not to reiterate the specific details in this framework, as this has already been done by Bohl and van den Bos (2012). Rather, we briefly emphasize several contributions this framework provides to social cognition in order to gain a more holistic understanding that can contribute to future directions in the field.

First, this framework leverages dual-process theories to aid in explaining social cognition. Cognitive psychology has rich history of research in this area going back decades (Schneider and Shiffrin, 1977; Shiffrin and Schneider, 1977; Kahneman, 2011). Briefly, dual-process theories argue that humans possess two main types of information processing mechanisms (see Birnboim, 2003; Evans, 2008; Evans and Stanovich, 2013). Automatic and stimulus-driven processing of information comprises Type 1 processing, while Type 2 processing is thought to be more controlled, cognitively flexible, and reflective. From an evolutionary perspective, Type 1 processing may have arisen earlier than Type 2 processing. These two processing types follow different pathways evident at the neural and functional levels, and emergent at the personal level (Bargh, 1984; Chaiken and Trope, 1999; Satpute and Lieberman, 2006; Lieberman, 2007; Frith and Frith, 2008, 2012; Adolphs, 2009). This distinction between automatic and controlled processing of information has provided a useful framework for understanding cognition generally (Birnboim, 2003; Adolphs, 2009), with researchers beginning to utilize this framework to better understand social cognition in particular (e.g., Frith and Frith, 2008; Bohl and van den Bos, 2012; Wilkinson and Ball, 2013). In line with dual-processing accounts, recent evidence in support of neurally dissociable perceptual and cognitive systems at play during social cognition was found when juxtaposing images and text in the processing of emotion (Sessa et al., 2014).

Accordingly, Bohl and van den Bos (2012) have proposed that direct perception of mental states is processed by Type 1 cognition, while the processes proposed by TT and ST occur as a result of Type 2 cognitive processes. These processes function in an interdependent and reciprocal fashion at the sub-personal level (Figure 1, arrow 1), but differentially express themselves at the personal level (arrows 3, 4). In line with one of the critiques we summarized earlier, they further propose that second-person research would allow for a more complete understanding of the roles of Type 1 and Type 2 processing in social cognition, in contrast with the traditional third-person observer research. Finally, IT emphasizes that any account of social cognition must extend beyond the individual level to that of the supra-individual level in which crucial factors that directly influence the sub-personal level such as interaction, context, and environment are considered (see Figure 1, arrow 2).

## Embodied, embedded, and enactive direct perception

Thus far, we have provided a high-level summary of social cognition research. The theoretical positions of TT and ST articulate evolutionarily advanced, extra-perceptual cognitive mechanisms (i.e., theoretical inference and mental simulation) that facilitate mental state understanding (e.g., Gopnik and Wellman, 1992; Blakemore and Decety, 2001) and successful interaction. These approaches to social cognition research have, however, been relatively neglectful of social cognition during interaction. But social interaction features prominently in IT. Social information perceived during interactions allows for the possibility of automatic and direct understanding of the mental states of another agent (e.g., Gallagher and Varga, 2013). And, as shown in our brief review of Bohl and van den Bos (2012), the different mechanisms posited by TT, ST, and IT can be integrated into a single overarching framework of social cognition by aligning their proposed functions and utilities with those of two types of cognitive processing from cognitive psychology and neuroscience.

It is our view that Bohl and van den Bos’ (2012) integrative framework of social cognition serves as a promising start, and one that we wish to further extend. Our extension centers on what we see as a conflation of social cognition with social perception. The former refers to the pattern of judgments, attributions and inferences we make about the motivations and mental states of others while the latter refers to our perception of the behavior of others. These processes are obviously inextricably linked, and, as has been argued elsewhere (Wilson and Golonka, 2013), any theory of cognition must start with a description of the informational basis for perception. A theory of social perception must, therefore, begin with a description of the information available for the perception of others, including their mental states. This represents a crucial distinction not yet accounted for by frameworks of social cognition, particularly those that include direct perception such as Bohl and van den Bos (2012).

The question then becomes: what social phenomena can be directly perceived and how might this occur? In answer to this question, we next turn to what we see as two complementary notions of direct perception: direct perception stemming from ecological psychology in embodied and embedded accounts of cognition (Chemero, 2009) and direct perception in enactive views of cognition (De Jaegher, 2009; Gallagher and Varga, 2013). Our goal, then, is to articulate an account of social perception starting with the informational basis of what can be directly perceived and articulating the ways in which this supports and informs other social cognitive mechanisms. In doing so, we provide a more detailed articulation of potential underlying mechanisms for social cognition, by offering an empirical means for reconciling radical and non-radical views of cognition. Towards that end, we turn next to a discussion of the embodied and embedded as well as enactive approaches to perception. This lays the foundation for a set of theoretical propositions meant to guide social cognition research.

### Embodied and embedded direct perception

Understanding the embodied and embedded approach to perception, action, and cognition, drawing from the ecological perspective, serves to enrich our understanding of not only the contributions gained from integrating the interactionist and ToM approaches to social cognition, but also the mutuality of an organism-environment systemic relationship (Marsh et al., 2006). From this perspective, human nervous systems, brains, and cognition are evolved adaptations that serve the purpose of planning and executing coordinated and goal-oriented actions with both the physical and social environment (Glenberg, 1997; Kaschak and Maner, 2009). Viewing cognition as an adaptation elucidates the notion that it is “shaped by a dynamic interplay among the nature of one’s nervous system, the nature of the environment in which one lives, and the manner in which one’s body can move in that environment” (Kaschak and Maner, 2009, p. 1237). From the ecological perspective, cognition evolved from a need to “coordinate and integrate increasingly complex behavioral repertoires across time and space” (Marsh et al., 2009a, p. 321) and that the distinction between perception, action, and cognition is an arbitrary one (Anderson et al., 2012).

Through an organism’s embodiment it is considered closely coupled with, not only its physical environment, but also its social environment (Anderson, 2005). On the embodied and embedded account, much of the information that an organism needs to engage in adaptive action is available within the environment and thus, does not always need to be mentally represented, as cognitivists propose (Brooks, 1991, 1999; Chemero, 2013). On this account, the necessary information is in the environment and, therefore, does not need to be represented. Perception, in such accounts, is thought of as direct. *Embodied and embedded direct perception*, then, is perception that is non-inferential, does not rely on mental representations, and is not always accurate (Chemero, 2009). This means that an organism directly perceives its environment as inherently meaning-laden in terms of what it can do in that environment; that is, an organism directly perceives *affordances* (i.e., opportunities for action; Gibson, 1979).

The notion of an affordance is a central concept in Gibson’s theory of direct perception. In these terms, the function of perception is to enable action, and this is achieved by the direct perception of objects and surfaces in terms of the actions that can be performed with those objects. Affordances are thus necessarily a relationship between the perceiving agent and her environment; they reflect a reciprocity (Lombardo, 1987) between the action capability of the perceiver and the properties of objects in the environment. On this account, affordances are directly perceivable because there is information available to an organism’s perceptual systems that specify that affordance with respect to the abilities of the perceiving organism (Marsh et al., 2009b). *Information* is defined as a pattern in ambient stimulation (e.g., the ambient optic array) that is always present when, and only when, the affordance is present. Because such patterns remain across changes in viewing conditions, such as distance, viewing angle, or illumination levels, they are said to remain invariant across transformations.

While much research on affordances emphasizes interaction with physical aspects of the environment, given that humans are fundamentally embodied and embedded in both a physical and social environment, Gibson’s theory of affordances is relevant for understanding social processes (e.g., McArthur and Baron, 1983; Zebrowitz and Collins, 1997). In fact, Gibson even noted that, “other animals afford above all, a rich and complex set of interactions, sexual, predatory, nurturing, fighting, playing, cooperating, and communicating” (Gibson, 1979, p. 128). It is our contention that *social affordances* can thus be taken to mean the opportunities for social interaction presented by verbal and non-verbal social behaviors as well as opportunities for coordinative action not available to an individual alone (McArthur and Baron, 1983; Valenti and Good, 1991; Kono, 2009).

This theoretical perspective has been empirically examined in a variety of domains. Examples of social affordances include: the alteration of the kinematic behavior during a feeding paradigm as a function of one interactor requesting to be fed through the opening of the mouth or direction of gaze (Ferri et al., 2011), reach-to-grasp kinematics as a function of manipulation of object relationship and interactor grip and gaze type (Scorolli et al., 2014), transitioning from moving planks of wood individually to moving the planks with another as a function of the scale of the plank relative to the collective action possibility of the two individuals together (Richardson et al., 2007; Isenhower et al., 2010), judging the aperture width for which an adult-child dyad can pass through on the basis of the scaled body dimension of the dyad as a whole (Chang et al., 2009), and the synchronous exchange of multi-modal behaviors such as facial expressions and gestures during a collaborative task (Louwerse et al., 2012). As such, social affordances provide insight into ways which humans engage in embodied cooperation and execute joint coordinative actions toward shared goals (Baron and Boudreau, 1987).

This conceptualization of social affordances can be characterized as that which enables a joint coupling of perception-action systems of more than one individual (Valenti and Good, 1991). Indeed, there is an increasing trend in the cognitive sciences to study the emergent social interaction dynamics that occur *in the world* rather than the cognitive processing occurring *in the head* (e.g., Marsh et al., 2006; Barrett et al., 2007; De Jaegher, 2009; Marsh et al., 2009b; De Jaegher et al., 2010). Accordingly, the types of social interaction afforded by a given situation are governed by dynamic laws that constrain motor systems of the body, the coupling of perception-action systems, as well as the coupling of person and environment (Good, 2007; Marsh et al., 2009a). Social interaction dynamics, then, “reflect a continuous flow of adjustments in the actions of one individual in response to perceiving the actions of the other and the flow of information from the individual’s own movement” (Marsh et al., 2006, p. 8).

In sum, social affordances and social interaction dynamics are the first component of our framework for integrating social cognition. In adopting this view of direct perception, we contribute to the aforementioned framework of social cognition by providing a level of specificity at the supra-individual level not detailed by Bohl and van den Bos (2012). We argue that, in the embodied and embedded account, direct perception is of social affordances and perceiving such action opportunities does not necessitate any mental state understanding between interactors (Marsh et al., 2009b). This is not to say that mental state understanding is not important for social interaction; rather, it is meant to draw attention to the interconnectivity of all the bodily systems involved when engaging in joint action with others (Marsh et al., 2009b). As we have alluded to before, social interaction is inherently complex and dynamic. Therefore according to this account, social interaction is driven, at least in cases of joint action and embodied cooperation, by meaning-laden information available in the physical and social environment. While this account of direct perception provides insight with regard to perceiving interaction possibilities without a need for mental state understanding, we turn next to the enactive account of direct perception that provides us with insight into how others’ mental states may be perceived.

### Enactive direct perception

One of the major critiques regarding Bohl and van den Bos’ (2012) integrative framework is that it conflates interactionism with enactivism (De Jaegher and Di Paolo, 2013). In enactivism, the experience of perception is not one that occurs solely in one’s head; rather, perceptions are enacted or actively brought forth through an organism’s engagement and sensorimotor exploration of its environment (Varela et al., 1991; Froese and Ziemke, 2009). In other words, an organism is not a passive receiver of information who must translate environments into internal representations and then add meaning or value to said perceptions; instead, through an active sense-making process, the world is perceived as meaningful (De Jaegher and Di Paolo, 2007). The enactive view of perception differs from the embodied and embedded view in that perception of invariant information relies upon specific motor actions and these actions, themselves, constitute the said perception (see Noë, 2004; Mossio and Taraborelli, 2008; Barrett, 2011). In other words, while the two approaches to perception share many similarities, the key difference resides in the fact that the enactive view incorporates specific neural predictions suggesting that direct perception is the persistent linkage of *both* sensory information with motor activations for a given stimulus (Mossio and Taraborelli, 2008).

Specific to the study of social cognition in enactivism is the notion of *participatory sense-making*. This construct is encapsulated by the idea that social interaction is central to social cognition. The enactive view uses this construct to describe the process of how social meaning is mutually constructed through interactions and is affected by patterns of coordination, as well as the breakdowns and recoveries inherent to the interaction (De Jaegher and Di Paolo, 2007, 2013; De Jaegher, 2009). For example, imagine a case where a manager calls her assistant into her office because a report did not get sent out in time. The tone of voice and facial expression of the manager informs the assistant that something is wrong. The manager inquires as to why the report was not sent and the assistant recognizes what was wrong. But, a response indicative of defensive behaviors is conveyed by the assistant. Through discussion, the assistant explains she did not recall being told the report needed to be sent out, rather, just completed by the date given by the manager. The manager realizes she was not clear enough in her prior instructions to the assistant. This brief example demonstrates participatory sense-making in that social meaning can be co-constructed during social interaction.

*Enactive direct perception* is critical to participatory sense-making and suggests that, when an individual encounters some other, they can “have a direct perceptual grasp of the other person’s intentions, feeling, etc.” (Gallagher, 2008, p. 535). Specifically, the perceptually available information provided by a person’s motor movements, expressions on their face, as well as their gestures, at least in normal, everyday contexts, is enough to allow for social understanding to be achieved (Gallagher, 2008; De Jaegher, 2009). The theorizing of Gallagher (2008) and De Jaegher (2009) have been instrumental in articulating an enactive account of direct social perception, but Gallagher and Varga (2013) provide the most concise and compelling argument. In this account, Gallagher and Varga describe direct social perception in a way that complements and elaborates upon the definition given in ecological psychology. In their account, *social perception is enactive* meaning that perception of others’ actions is in terms of responses to those actions and not necessarily in terms of their mental states. Direct perception, in this case, is not just a sensory process, but also a preparedness for action that includes the supporting sub-personal processes (neural activations). The addition of these sub-personal processes, on this account, does not add an inferential component to perception; rather, it constitutes the meaning, and not just the surface features, of what is perceived for purposes of engaging in interaction (see Gallagher, 2008; Gallagher and Varga, 2013).

While they note that enactive direct perception is in terms of responses to other’s action and not in terms of mental states, Gallagher and Varga (2013) make a compelling case for the direct perception of certain intentions and emotions conveyed by other people; mental states that have often been argued to not be available for perception. They note that motor and proximal “intentions are in the movement, in the action, in the environmentally attuned responses” (p. 6) and thus, there is a perceivable intentionality because it is evident in the embodiment of others (see also Gangopadhyay and Schilbach, 2012). In other words, during interaction, we can easily see the intentions of others through the actions they take and the associated bodily movements with said actions. The authors support this claim by referring to research showing that varying types of intentions are discernable when viewing others’ actions, and even when viewing decontextualized actions in the form of point-light displays, often with a high-level of accuracy (see Sartori et al., 2011; Becchio et al., 2012).

Further, the authors posit that, because emotions manifest in physical and behavioral cues, and thus embodied by the individual expressing them (see also Niedenthal, 2007), emotions too may be available to direct social perception. In such cases, the authors point out that direct perception of emotion is subserved by information. In this way, they acknowledge the importance of situational and environmental cues as well in accurately perceiving emotions and thus, scaffolding the perception of complex emotions (see Gallagher and Varga, 2013, pp. 6–7). Of course, when the direct perception of emotion relies on numerous cues that are “free to vary” it becomes difficult to say when and under which circumstances emotions are directly perceived. This, too, points to the need for a better understanding of social information in any account of social cognition.

### Complementary notions of direct perception

Both the embodied and embedded direct perception and enactive direct perception represent the second component of our framework for integrating social cognition. These are congruent in that they provide an account of perception in terms of social interaction possibilities. They differ in one primary aspect, however. The former focuses on the interaction dynamics specified by invariant information provided by some social other, often for purposes of engaging in joint and coordinative action. The latter complements this by positing that the mental states (i.e., certain intentions and emotions) and motor activations are a part of the information directly perceived, even if what is perceived is not explicitly in terms of mental states (e.g., Chemero, 2009; McGann, 2014). Put more succinctly, the integration of these two views of direct perception means that there may be both kinematic information as well as other behavioral cues directly indicative of mental states available for perception. To bolster this argument, we next provide an account of the informational basis for social perception that allows us to better integrate these complementary notions of direct perception.

## Informational basis for social perception

To better understand social cognition generally, and direct social perception in particular, we next provide a tentative outline of an informational basis for social perceptions. In accordance with Gibson’s (1979) theory of direct perception (see also Turvey et al., 1981), information must take the form of an invariant pattern in some medium to which the human perceptual systems are sensitive, and that lawfully corresponds to some state of affairs in the world. We start by reviewing research and theory related to the kinematic specification of dynamics (KSD) principle and elaborate on this idea with what we term the social cues and signals distinction. Within this framework, we highlight a number of relevant points that enrich our discussion of the informational basis for social perception. From this, we offer a set of testable propositions designed to further research on the boundaries of direct social perception.

### Kinematic specification of dynamics

We begin a tentative informational theory of social perception by borrowing the principle of KSD (Runeson and Frykholm, 1981, 1983). According to the KSD principle, events in the world have causes which lead to characteristic kinematic patterns. There is, by natural law, a 1:1 mapping between the movement kinematics of an event and its underlying cause.

This idea has not been without criticism, and the origins of the controversy lie with Hume’s treatment of causality. In Hume’s argument, we cannot know about the cause of an event, only its motions. For example, when a rolling billiard ball strikes another, and the second ball rolls away, Hume argues we only see the motion of the first followed by the motion of the second, and that the underlying causal force is not seen, and thus cannot be known. Since then, psychology has mostly relied upon a cognitivist solution to the problem of causality. Beginning with Kant’s invocation of innate categories of thought, spanning to Helmholtz’s unconscious inference and beyond, perceptionists have generally agreed that the cause is not given in the senses and must somehow be inferred. It was not until Michotte’s (1963) groundbreaking work on the perception of causal events that anyone took seriously the possibility that causal information might be given after all. In Michotte’s experiments, observers were shown simulations of novel collision events, in which the timing of the collision varied systematically—specifically the length of time the two colliding objects were in contact with each other and how they moved while attached. Michotte found that observers recognized collision events when the timing was appropriate to a real collision, and spontaneously applied other names to the other events with different timing (see Twardy and Bingham, 2002, for a similar demonstration of observer sensitivity to violations between kinematic patterns and their underlying dynamics). In contrast to Hume, Michotte concluded that such performance could not be based on passive associations from past experience, because of the novelty of the events, and must therefore involve identification of the event itself.

Later, the work of Johansson (1973) laid the foundation of modern event perception work when he created the patch-light technique by attaching small lights to the major joints of human actors and filmed them walking in the dark. This showed that observers had little difficulty recognizing the events as humans walking. In one study (Johansson, 1976), he showed observers short video clips of human patch-light walkers, and similar displays of marionette puppets engaged in the same action. Despite the similarity in spatial patterns of the movements (i.e., both displays depicted the same major joints moving in the same directions relative to each other), observers could easily distinguish the biological motion from the non-biological, even when the video clips were less than half a second in duration.

Afterward, multiple studies have demonstrated the wealth of information that observers are able to perceive in displays of patch-light actors, including the sex of the actor (Kozlowski and Cutting, 1977), identity (Cutting and Kozlowski, 1977; Loula et al., 2005), the amount of lifted weight (Runeson and Frykholm, 1981), emotion (Dittrich et al., 1996; Pollick et al., 2001; Atkinson et al., 2004), and even deceptive intent (Runeson and Frykholm, 1983). In the series of studies conducted by Runeson and Frykholm (1983), they filmed patch-light displays of actors pretending to make a light box seem heavy when lifted. Not only were observers able to detect the deception, they were able to correctly judge the weight of the box. In another variation, Runeson and Frykholm asked the actors to act like the opposite gender. Again, observers were able to recognize the deception, correctly recognizing both the actor’s true gender, and the fact that they were mimicking the other gender. The researchers concluded that “perceiving another person has two aspects: true properties of person and action, and intentional or communicative expressions (Runeson and Frykholm, 1983, p. 610)”. In their analysis, information for both aspects is available to the visual system, and both are separately perceived.

These studies led Runeson and Frykholm (1983) to present a theory of person and event perception based on the KSD principle. The underlying causal dynamics of an event generate a unique kinematic pattern of motion in that event. Perception of movement kinematics thus reveals the underlying dynamics (i.e., motions specify their causes). Variations in the center of gravity between the sexes create different movement dynamics in men and women, which then leads to differences in the spatio-temporal pattern of points in patch-light displays. Variations of weight in a lifted box alter the center of gravity of the lifter, leading to differences in the spatio-temporal patterns of points in patch-light displays for lifters of light vs. heavy boxes. One may attempt to make a light box look heavy, most likely by moving slower than is necessary and incorporating the lower limbs, but these deceptive movements cannot alter the load on the body imposed by the weight.

While later studies have continued to investigate the nature of the patterns of motion that specify the causal event (e.g., Bingham, 1987; Bingham et al., 1995; Muchisky and Bingham, 2002; Wickelgren and Bingham, 2004, 2008; Bingham and Wickelgren, 2008), this work has focused on mechanical events (e.g., pendulums and other oscillatory events). While traditional mechanics can be employed to understand the nature of the mapping from dynamics to kinematics in such events, there is as yet little understanding of how intentions, as a dynamic cause of behavior, map to kinematics, and whether such mappings are unique and count as information. However, the work of Becchio et al. (2012) is making headway in specifying the types of intentions that can be perceived based on kinematics. These authors claim that intention constrains the kinematics of a reach-to-grasp movement, citing studies that found differences in grip aperture and closing velocity as a function of the social intent of the action (Becchio et al., 2008, 2010; Sartori et al., 2011). Planning a reach involves selecting the appropriate movement kinematics to achieve a goal, and thus must reflect the prior intention; that is, *why* the agent is reaching. Other studies then demonstrated that, based on just kinematic information available in patch-light displays, observers were able to discriminate between reaches guided by different intentions (e.g., Manera et al., 2011). Thus, the theory that intentional dynamics can and do constrain movement kinematics, and that such movements can, in principle, specify their causes, is reasonable and has tentative empirical support. This must be the case if one were to claim, as we and others do, that intentions are embodied (Runeson and Frykholm, 1983; Mark, 2007; Gangopadhyay and Schilbach, 2012).

What remains then is to solve the problem of intentional dynamics (e.g., Shaw and Kinsella-Shaw, 1988). A complete KSD theory for social perception must address the issue of what intentions are, and how they shape action. The work of Shaw and Kinsella-Shaw (1988) has attempted to map out these questions, as they have endeavored to develop an ecological mechanics to account for the natural laws of goal-directed behavior. In this view, intentions are not defined in a teleological sense (i.e., as an assumed purpose behind any act), but as a mathematical operator that governs goal-selection. Michaels (2003) has speculated that such intentional dynamics can be used to define affordances as quantities conserved “over the space-time interval between goal-selection and goal achievement (p. 145).”

While there is much yet to be explained in this regard, KSD represents the third component of our framework for integrating social cognition. The point here is to emphasize that optical information about others can exist, even in reduced viewing conditions, such as patch-light displays. But, as most know through their familiarity with social interaction, there is often a plethora of information provided by a given social situation and, whether through perceptual or cognitive processes, meaning is ascertained. This leads us to the next part of our account on the informational basis of social perception: the distinction between social cues and social signals.

### Social cues and social signals

The social cues and social signals distinction (Vinciarelli et al., 2009) has recently been employed in attempts to translate insights from human social cognition to better understand the social dynamics of human-robot interaction (Fiore et al., 2013; Wiltshire et al., 2013, 2014b). However, it is also of general utility for understanding human social cognition and, in particular, detailing our account of the informational basis for social perception. In line with embodied and embedded views of social psychology (e.g., Marsh et al., 2009a), we suggest that social cues and social signals provide the basis for linkage between the theories that allow for an explication of social meaning spanning the environment, the body, and the brain (Streater et al., 2012; Lobato et al., 2013; Wiltshire et al., 2014b).

At a fundamental level, we take the position that there are physical and behavioral cues that comprise *social cues*, all of which are available to an organism’s perceptual system in the environment and serve as channels of potentially useful information. *Physical cues*, then, are those cues that convey aspects of an organism’s physical appearance as well as environmental and situational factors that are socially salient. Examples of a physical cue might be the type of clothing worn by a person or the relative distance that that person may be from the person perceiving them. By contrast, *behavioral cues*, then, are those cues comprised by the “non-verbal movements, actions, and gestures as well as verbal vocalizations and expressions using the body and face” (Fiore et al., 2013, p. 2). In this way, physical cues are often viewed discretely whereas behavioral cues tend to unfold dynamically. Of course, situations and environment, too, can unfold dynamically, but this is likely over a larger temporal scale than behavioral cues. Consistent with these papers, the term cue is not used here in the same sense as it is used within the literature on direct perception to indicate a sensory datum that requires inferential processing to ascertain meaning. Rather, social cues are any data available to a perceptual system that may support either inferential or direct perception.

In a previous account, we noted that social signals are in essence the meaningful interpretations of social cues as a function of the mental states and attitudes attributed to an agent that displayed them (Fiore et al., 2013). However, in light of the current discussion (i.e., to determine what social information may be directly perceivable to humans), we refine this to suggest that a *social signal* can be defined as the perceived intention or mental state comprised by the combination of observed social cues. This refinement leads to a question fundamental to this line of thinking: *can social signals (the meaning of the cues) be observable in the same way as social cues?* It is our view, and others, that it is possible to gain direct access to social meaning (cf. Zebrowitz and Collins, 1997; Marsh et al., 2009a; Gallagher and Varga, 2013), at least under some circumstances, and in others, perhaps not (e.g., Freeman and Ambady, 2011). That is, as alluded to previously, our aim is to provide further elaboration on accounts of direct perception, but to also attempt to express the limitations of what may be directly perceived and must therefore be inferred, at least with regard to mental states experienced in the social environment.

Drawing from the KSD principle, we argue that, in some cases, social cues can be thought of as the kinematic movement (especially behavioral cues) and the social signal can be thought of as the underlying dynamic cause. In other words, mental states such as intentions and emotions are linked to the motor system in such a way that their presence is evident in motion kinematics (Mark, 2007). If this is the case, then there should be a 1:1 mapping between social cue(s) and a social signal (Runeson and Frykholm, 1983). Likewise, in accordance with theories of direct perception, perceiving information comes with knowledge of a state of affairs in the world, when information is defined as a lawful relationship between the information and the real-world (Turvey et al., 1981; Turvey, 1992). This is an important distinction because if such relationships exist, then social signals must be treated as directly knowable, and as such, observable. In other words, a definable and measurable relationship between social cues and signals would provide support for the idea that another’s mental states are directly observable. That being said, some clarification with regard to this relationship is needed. A social signal is directly perceived only when the structure of an invariant set of cues yields perception without any need for probabilistic inference (Zebrowitz and Collins, 1997). We therefore propose the following:

*Proposition 1*. A lawful mapping between socials cues and social signals would render social signals as observable in the environment and lend support to accounts of direct social perception only if the set of social cues remains invariant as a function of their invariant association with a particular social signal. That is, across situations where specific actors and contexts vary, yet social signals remain constant, there would be also an invariant pattern of social cues that support social perceptual constancy.

As noted, the social environment is inherently complex and dynamic. Social cues can constellate in many combinations and their meaning (the signals) is contextually and culturally determined (Fiore et al., 2013). The process of understanding a person occurs in real time and perhaps, a barrage of social cues can be conveyed over short durations of time, some of which can be conflicting (i.e., “sending mixed signals”). Because different social cues are conveyed moment by moment, it can be difficult to attribute meaning to social agents, when perhaps the cues being displayed do not map neatly to one particular signal (cf. Freeman and Ambady, 2011). This, in turn, warrants we mention debates surrounding accuracy in social perception (see Kruglanski, 1989; Fiske, 1993; Jussim and Zanna, 2005; for reviews). As noted, direct perception does not need to be accurate (Chemero, 2009), but as complexity increases in the number and combinations of cues, so too, does the likelihood of making inaccurate perceptions of the social signals (see Doherty and Kurz, 1996; Wiltshire et al., 2014b). Therefore, in cases where the set of social cues is not invariant and naturally specifying the social signal, it is likely that the perception of the social signal will be probabilistic (Jussim and Zanna, 2005). We thus propose the following:

*Proposition 2*. For cases where a mapping between social cues and social signals does not exist, the interpretation of such cues must necessarily be probabilistic and therefore, relies on extra-perceptual processes rendering the social signal unobservable.

While future empirical work will need to differentiate which social signals are directly perceivable and which ones are not, we suggest that motor intentions, present intentions, and basic emotional states are the most likely candidates (see Gallagher and Varga, 2013). While some research has attempted to examine direct perception of dispositional features such as personality traits (e.g., Zebrowitz and Collins, 1997), we suggest that states are more likely candidates for direct perception and traits likely require extra-perceptual processes. More generally, in bolstering the argument for these propositions, elaboration of Chemero (2009) theory of information for direct perception and the distinctions between projectable and non-projectable properties are discussed to complement our social cues and social signals framework. In much the same way as how optic flow research has provided geometric descriptions of the information potentially available to perceivers (e.g., Nakayama and Loomis, 1974; Lee, 1976), our point is that research should here strive to describe the actions of others and provide a kinematic and geometric analysis of higher-order invariant patterns that can, in principle, work as the social cues that specify social signals.

### Situation semantic perspective of information

What is important to our argument is the notion that cognition is socially situated (see Smith and Semin, 2007). One of the fundamental ideas of this view is the fact that situations play a foundational role in cognition and that cognition is often for the purposes of adaptive social interaction (see Semin and Smith, 2013 for review). While a detailed argument for Chemero’s (2009) situation semantic view of information can be found in his book, our purpose for reviewing it here is to discuss how it relates to our social cues and signals framework and the above propositions. The situation semantic view of information dates back to Barwise and Perry (1981), Barwise and Perry (1983) and posits that information is a part of the natural world that exists in situations.

The underlying idea is that the meaning of a situation can be carried by a token of that situation and this token, in turn, carries information about a related situation if that situation constrains the present situation (Chemero, 2009). On this view, the idea that tokens can specify something else (e.g., the meaning of the situation or other situations) is akin to what we posit with regard to social cues and signals. That is, a social cue, or a set of cues, can specify the meaning of a current social situation, a certain interaction possibility, or perhaps, the mental states of others. Further, the situation semantic view not only allows for information to specify meaning through natural law, but also to indicate conventions which may not be underwritten by natural law (Turvey and Carello, 1985). We expect that further elaboration in this vein might be fruitful for advancing this work given that this ecologically sound perspective of information seems to leave room for what might be construed of as direct and indirect perception. Next, the distinction between projectable and non-projectable properties, stemming from embodied yet representational approaches to perception, provide further elaboration of these points.

### Projectable and non-projectable properties

The distinction between projectable and non-projectable properties helps to further articulate cases in which social signals might be directly perceived. The distinction, to our knowledge, was first argued for in Epstein’s (1993) representational account of perceptual systems and later reiterated in embodied accounts of memory (Glenberg, 1997) and social cognition (Kaschak and Maner, 2009). The general idea is that the environment contains *projectable properties* that are objective features readily and directly available for perceiving (Epstein, 1993; Kaschak and Maner, 2009). Regarding a physical environment, this would include, but is certainly not limited to, the spatial structuring of the environment as well as the form, shape, and color of objects. At a more general level, projectable properties are those that are specifiable by light projected on to an organism’s optic array (Epstein, 1993; Glenberg, 1997). On this account, there are also, *non-projectable properties* of the environment that are not readily available to perceptual systems, and thus require further mental operations in order to glean the meaning of such properties (Kaschak and Maner, 2009). An example of a non-projectable property is object ownership (Schmidt, 2007), which Glenberg (1997) argues can only be determined by drawing from the memory of prior experiences and not from direct perception.

Our position, however, diverges from Kaschak and Maner at the point that they provide their example of what is projectable and non-projectable in the social environment. But, it is important to note this difference in perspective here. With regards to the social environment, Kaschak and Maner (2009) use the example of gaze following, wherein the orientation of the face and eyes are projectable properties; however, the notion that the eyes are *looking at and perceiving* *something* is a non-projectable property (see also Adams and Kleck, 2003). On our view, orientation of the face and orientation of the eyes might represent two social cues, and the fact that these cues are likely an invariant set of cues signaling that the person is looking at and perceiving something leads us to argue that this is directly perceivable.

The point here is not to argue this particular case of social cues and signals, or any other for that matter. Instead, our point here is to articulate this useful distinction as it appears to be necessary for understanding what information is available for direct social perception. Projectable properties are directly perceivable, but they may or may not actually be perceived, depending on perceptual learning and attunement. In other words, detection and use is something that requires attunement and calibration (Bingham et al., 2001) and this likely applies to the social environment as well. *Perceptual learning* is the process of discriminating opportunities for action from distinctive features of the environment and invariant relationships as a function of the motor repertoire of the embodied organism and reciprocally assigning salience to environmental features (McArthur and Baron, 1983; Gibson, 2000; Pezzulo et al., 2011). Though beyond the scope of this paper, some suggest that this process is explained by neural networks and their ability to accrue experience and attune to changes in cues and features of the environment that occur over time (e.g., Keysers and Perrett, 2004; Barrett et al., 2007). Regardless, over time, perceptual learning and attunement occur to allow for the affordances of projectable properties of the social environment to become directly perceivable provided they afford a particular action or interaction (Gibson, 2000; Barrett and Kurzban, 2006) or perhaps, as enactivists might argue, others’ mental states (De Jaegher, 2009; Gallagher and Varga, 2013). Summarizing this, and framing it in the terms of social cues and signals, we propose:

*Proposition 3*. All social cues are projectable properties and therefore, some social signals can be directly perceived upon first encounter *if* the cue set for that signal is invariant; whereas, there are other social signals that, while they may not be immediately perceived, can be through perceptual learning, attunement, and calibration because they are comprised by projectable properties.

By contrast, non-projectable properties are not directly perceivable^3^. Non-projectable properties can, perhaps, only be detected indirectly using inferential mechanisms or, as Epstein (1993) argues, be represented. We remain agnostic to the notion of representation here. But, it would seem that non-projectable properties of the social environment, therefore, can only be inferentially perceived and, thus, according to Kaschak and Maner (2009), require social interaction and the process of teaching and learning in order to detect. One possibility is that more simple forms of social cognition, such as brief interactions with strangers, may rely almost exclusively on projectable properties; whereas, relatively more complex forms of social interaction (e.g., involving the need to think about combinations of relationships; Fiske, 2012), may draw more heavily on non-projectable properties. In our terms, we can then propose:

*Proposition 4*. Even though all social cues are projectable properties, some social signals are non-projectable and can thus never be directly perceived. In such cases, more complex extra-perceptual mechanisms are required to interpret the social signal conveyed by social cues and this may be done through inferential, representational, simulative, or perhaps other mechanisms.

While only briefly elaborated upon here, social cues and social signals represent the fourth component of our framework for integrating social cognition. The ability to perceive projectable properties and infer non-projectable properties of stimuli in the environment has provided humans with an extended ability for adaptive and flexible interaction with the environment. This is an essential social cognitive capacity given the extent to which the environments in which humans operate are fundamentally social in nature (Kaschak and Maner, 2009). As is currently known, humans exhibit the most complex social activity observed spanning varying levels of social interaction, relations, and hierarchies (Barrett et al., 2007). Therefore, to us, it seems plausible that, because social information is so complex, it is not always directly perceivable. However, there appears to be evidence that the human perceptual system is equipped to directly perceive social information, in the same way that it is equipped to directly perceive physical information about the environment.

### Dorsal visual system contributions to direct perception

Norman (2002) reviewed psychological, neuroscientific, and neuropsychological findings on the ventral and dorsal visual systems in an attempt to draw parallels between the two visual systems and constructivist (i.e., inferential) and ecological accounts of perception. Relevant here, Norman (2002) proposed that a primary function of the dorsal system is to enable visually guided behavior in our everyday life (see also, Milner, 1998; Milner and Goodale, 2006; Binsted et al., 2007). Accordingly, the dorsal system processes visual information more quickly because the magnocellular visual pathway (M-pathway), which provides direct input to the dorsal system, has a higher temporal sensitivity. Further, the dorsal system does not necessarily result in conscious awareness of the perceived objects. This is supported by evidence from patients with damaged or destroyed ventral systems but intact dorsal systems who fail at identifying the perceptual qualities of objects (e.g., size, shape), but nonetheless enact appropriate motor programmes when interacting with those objects (e.g., accurate gripping of the object; Milner, 1998; James et al., 2003). Likewise, the dorsal system does not appear to rely on long-term representational memory, as the execution of actions only requires short-term storage of information. From this, Norman (2002) argued that the functions of dorsal system align well with ecological psychology’s theories of visual perception. Put another way, the dorsal system may be the visual system allowing for direct perception as argued by Gibson (1979). Though Norman’s primary focus was on how the ventral and dorsal systems process the *physical* properties of objects, we suggest that the same functions might allow for visually guided social behavior in response to processing *social cues and signals*. Norman characterizes the dorsal ventral system analogously to descriptions of Type 1 processes described earlier (see, Evans and Stanovich, 2013), making it an appealing candidate for integration into a framework of social cognition at the sub-personal level.

Subsequent research on the dorsal visual system provides some support for our suggestion. Rizzolatti and Matelli (2003) examined the anatomy of the dorsal visual system and argued that the dorsal stream is comprised of two visual streams: the dorso-dorsal stream, which guides behavioral control, and the ventro-dorsal stream, which plays a crucial role in the perception of space and action. The dorso-dorsal pathway runs from the superior parietal lobule to dorsal pre-motor areas, and governs control of online actions, while the ventro-dorsal pathway progresses from the medial superior temporal area to ventral areas of the pre-motor cortex, and is concerned with both the perception of space and action understanding. Studies of stroke patients have revealed that the posterior middle temporal gyrus is a critical region for associating action with meaning (Kalénine et al., 2010; see also, Binkofski and Buxbaum, 2013). Though this research has largely focused on understanding actions in relation to objects, such as tool use, we suggest it is possible that the same network plays a crucial role in understanding the meaning (i.e., the social signal) associated with generally observing social cues.

Although research on the role of the magnocellular pathway in processing social cues has been limited, we find further suggestive evidence in favor of the above hypothesis from studies on emotional facial expression. For example, there is evidence that the M-pathway quickly processes emotional facial expressions (Vuilleumier et al., 2003), as well as research showing M-pathway abnormalities in individuals with disorders where processing social information is impaired, such as schizophrenia-spectrum disorders (Bedwell et al., 2013) and autism spectrum disorders (Greenaway et al., 2013). The deficits in processing social information found in these disorders could be a consequence of a visual system, specifically the dorsal system components, with an impaired ability to directly perceive relevant social information. This emphasizes the potentially informative nature of investigating dorsal system processing of other social cues and signals. From this, we propose the following proposition:

*Proposition 5*: The ability to directly perceive opportunities for interaction or coordinative action (i.e., social affordances) is a function of the dorsal visual system’s contribution to social cognition. More specifically, the middle temporal gyrus functions as a supramodal neural region critical for transforming perceived social cues into meaningful social signals, though of course other neural regions are likely involved in this process (Anderson, 2010). A corollary of this hypothesis posits that individuals with a dysfunctional dorsal system would be impaired in directly perceiving social affordances due to an impaired ability to perceive social cues.

In the final section of our paper, we draw from, and elaborate upon the Brunswikian and Gibsonian integration proposed by Vicente (2003). Our goal is to make our case and provide the means through which we can more specifically define and measure the relationships between social cues and signals and thus, direct social perception.

## Toward an integrative account of social cognition

It is our view that to understand the mechanisms of social cognition, and develop a more integrated research approach, there is need to invoke explanatory pluralism (see Dale, 2008; Dale et al., 2009). While some are skeptical of integrative approaches (e.g., De Jaegher and Di Paolo, 2013), we align with the view of pluralism in cognitive science that a better understanding of the mechanisms of social cognition, or cognition more generally, requires the integration of competing theories into meta-level frameworks that sustain the co-existence of each (see Dale, 2008). Specifically, with the growing body of evidence for the various proposed mechanisms of social cognition and the different levels of explanation associated with each, it seems increasingly likely that humans may have the capacity to employ each of these mechanisms during the various types of engagement with the social environment (i.e., direct social perception, theoretical inference, and simulation). While the aforementioned review provided us with the theoretical scaffolding necessary to advance thinking in social cognition, what is required is a means to test the aforementioned propositions. Towards that end, we next elaborate on how Vincente’s (2003) integration of Gibson’s affordances with Brunswik’s Lens Model, provides us with the empirical scaffolding to push this research forward.

Vicente (2003) proposes a theoretical integration between Brunswik’s probabilistic Lens Model (see Brunswik (1956)) and Gibson’s, in Vicente’s words, more deterministic direct perception of affordances (see Gibson, 1979). The two approaches are both aligned in that they focus on the relation of an organism and its environment, but distinct in how perception of the environment is conceptualized.

Brunswik’s (1956) core premise is that of *probabilistic functionalism*. This means that, to understand how an organism accomplishes its goals (achievement), the aspects of that agent’s environment that it attends and responds to in order to attain those goals must be known (functionalism). In addition, Brunswik’s approach is committed to the idea of causal ambiguity, from which the relationship between environmental cues and an organism’s perception of them is uncertain and, therefore, likely to be probabilistic (probabilism; Doherty and Kurz, 1996). To better understand the rationale for probabilism, the model relies on the constructs of distal vs. proximal stimuli wherein, *distal stimuli* are objective states of an organism’s environment and *proximal stimuli* are the features of the environment perceivable to the organism (Vicente, 2003). According to Vicente (2003), “Brunswik believed that an organism is not able to perceive distal stimuli directly but instead must infer what is going on in the ecology from the imperfect (i.e., probabilistic) cues provided by proximal stimuli” (p. 246). This view is thus a more radical distinction than the projectable and non-projectable properties discussed above as this would, in essence, posit that no perception can be direct because it is always inferential.

By contrast, and as we have already discussed, Gibson (1979) believed that perception could be direct in that meaning could be perceived in the environment without the need for inferential mechanisms and this was captured by the notion that direct perception is of affordances. While many of the central tenets of Gibson’s view have been detailed, Vicente’s (2003) integration corroborates this when he notes perception can only be direct when there is a lawful mapping between an environmental invariant and an affordance and that this relationship is what counts as ecological information. Central to this is that direct perception is an active rather than a passive process. In other words, an organism is actively attuning to the information in the environment for the purposes of engaging in interaction with it.

Ultimately, what Vicente (2003) suggested was that Brunswik’s Lens model and Gibson’s Affordances are able to account for different, albeit, complementary, types of environmental interaction. Put simply, “direct perception requires invariants, whereas judgment and decision making require probabilistic cues” (Vicente, 2003, p. 259). Indeed, Gibson’s approach emphasizes perception-action coupling whereas Brunswik merely emphasized perception. These distinctions are important for our argument about social cognition, because the types of situations used in traditional social psychology experiments have required people to make passive judgments about social stimuli (cf. Good, 2007). As we noted earlier, others have either called for, or emphasized, the need for research to be conducted that examines more direct perception-action links like those occurring during actual social interactions and that do not necessitate more complex mental operations (Vicente, 2003).

In terms of our social cues and signals framework, it is our position that leveraging the theoretical integration provided by Vicente is necessary to advance this work. Indeed, we have recently elaborated on the use of the Lens Model in this context (Wiltshire et al., 2014b). A key insight highlighted in that work is that “Social Judgment Theory” (SJT; as detailed specifically by Cooksey, 1996; Doherty and Kurz, 1996), which heavily relies on Brunswik’s Lens Model, has not yet examined social judgments in the sense that traditional social cognitive researchers use it. For example, this would expand SJT research by asking participants to make a judgment about the intentions, emotions or other mental states of a target person. Related research has employed the Lens Model to examine the relationship between nonverbal cues and personality judgments (e.g., Gifford, 1994; Hirschmüller et al., 2013), but to the best of our knowledge, this work is distinct from what we propose here. Specifically, social cue and signal judgments get more at the immediate, underling intentionality or mental states whereas extant work examines more stable dispositions that may not map onto mental states at a given context-specific moment. Our novel contribution in reiterating this here is that, although the Lens Model is used as a technique for understanding the relationships between inherently probabilistic cues, we feel its use as an analytic scaffold will help to advance our argument with regard to the propositions proposed herein.

**Figure 2:**
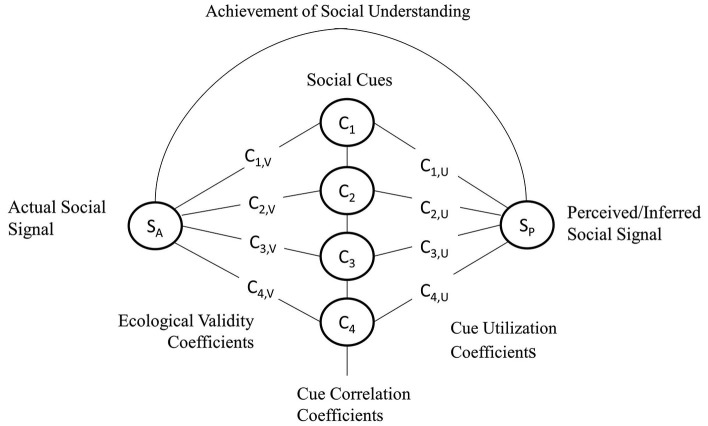
**A social cues and signals depiction of the Lens Model (Adapted from the works of Hammond, 1993; Cooksey, 1996; Doherty and Kurz, 1996; Vicente, 2003)**.

Figure 2 represents our repurposing of the Lens Model within the social cues and signals framework. Drawing from efforts in SJT (Hammond, 1993; Cooksey, 1996; Doherty and Kurz, 1996; Vicente, 2003), our version of the Lens Model specifies how perceptions or inferences of a social signal (S_P_) can be made with regard to the proximal and projectable social cues (C_i_) that are available to an organism’s perceptual systems. If, indeed, the perceived social signal (S_P_) matches the actual social signal (S_A_) then this would lead to the achievement of social understanding. As is tradition in SJT, the Lens Model serves as an analytic tool to decompose the relationships between the components of the model that ultimately represent a judgment process (see Cooksey, 1996). To do so, a number of values are needed. Ecological validity coefficients (C_i,V_) are the correlations between the actual social signal (S_P_) and the social cues (C_i_). In turn, the cue utilization coefficients (C_i,U_) are weights placed upon certain cues available to that agent in its perception or inference that a certain social signal indeed exists. Lastly, cue correlation coefficients represent the correlation between each of the available social cues (C_i_), but a variable is not used to represent these here because the correlation needed is factorial with regard to the number of cues. That is, a correlation coefficient is required, for example, for the relationship between C_1_ and C_2_, C_1_ and C_3_, C_1_ and C_4_, C_2_ and C_3_, C_2_ and C_4_, and C_3_ and C_4_.

In short, this version of the Lens Model is proposed for two purposes: to highlight a novel application of SJT (i.e., to study the relationship between social cues and signals), and to convey what might be necessary for direct social perception. This helps set the stage for empirical examination (see Wiltshire et al., 2014b for additional methodological issues). That is, the question then is how to define an invariant set of cues with a lawful relationship between the cues (C_i_) and the perceived or inferred social signal (S_P_) as argued for in Proposition 1. To address this, we propose:

*Proposition 6*. A lawful relationship between an invariant set of social cues and the perceived social signal would manifest through high cue correlation coefficients between all cues comprising the set, and equally high cue utilizations coefficients.

Note here we do not propose perfect correlations as would be expected by the 1:1 relationship proposed for direct perception (e.g., Vicente, 2003), nor do we provide a specific threshold for what constitutes “high correlations.” While this decision is open to critique, we would like to leave this issue as a point of discussion for empirical examination. But, perhaps, a relationship slightly less than 1:1 may enable direct perception; whereas, a relationship significantly less than 1:1 would require inference. To reiterate, Brunswik’s weighting system in the Lens Model can range from 0 (e.g., no ecological validity of a cue) to 1 (e.g., perfect ecological validity of a cue). Those weights, within some tolerance, but otherwise very nearly perfect, are thus lawful and support direct perception, whereas, we could conclude the remainder likely supports inference. As McGann (2014) recently argued, direct social perception needs to be sufficiently reliable to guide behavior in normal situations, but does not necessarily have to be exception free. This interpretation lends credence to our notion that direct perception may be possible without a 1:1 relationship.

Criticisms of using the Lens Model as an analytic technique to provide an index of direct social perception are, of course, to be expected. For example, Zebrowitz and Collins (1997) noted that the Lens Model is limited in the degree it can provide theoretically meaningful cue configurations. In addition to this, the Lens Model only captures a snapshot of social cognition *a posteriori*, when in fact, social cognition occurs dynamically in an ongoing stream of activity (McGann, 2014). Likewise, the interdependent processes of social perception and social cognition are dynamic and interactive and, in order for any model of this process to be ecologically valid, they must flexibly shift towards different potential meanings in real time as different social cues are conveyed (Freeman and Ambady, 2011). Additionally, another limitation of the Lens Model is its artificial isolation of sender and receiver in a social interaction. The inherent meaning of an interaction resides ultimately in the relationship between two or more agents who are both senders and receivers as they interact over time. Therefore, to clarify, we propose this version of the Lens Model here as a theoretical and analytic scaffold to structure our argument and as a means to tractably test our propositions on the relationships between social cues and signals, but do not claim that the model proposed here fully captures the complexity of social dynamics.

**Figure 3:**
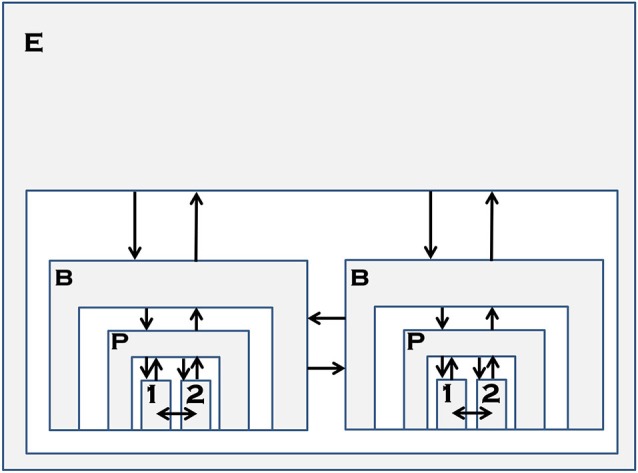
**A dynamic representation of social cognition highlighting interdependency and reciprocal influence across the regions of the nervous system associated with Type 1 or Type 2 processes, personal-level experiences (P), bodies (B), and their coupling with each other and the **(E)** environment (Adapted from the work of Beer, 2000; Bohl and van den Bos, 2012; Froese et al., 2013)**.

At a fundamental level, “social systems are dynamical systems” and, thus, the study of such systems should be treated accordingly (Richardson et al., 2014, p. 251). Indeed, research on direct perception typically relies on the theories and tools of dynamical systems (e.g., Chemero, 2009; see Richardson et al., 2014 for review). The dynamical systems perspective and associated methodologies are of great utility here as these provide a means of characterizing social interaction and the accompanying social cognitive processes across sub-personal, personal, and supra-individual levels. In the terms of this approach, examination of these levels as an integrated unit of analysis is the study of an organism-environment system (Järvilehto, 1998), which has previously been extended to characterize social phenomena (e.g., Vallacher et al., 1994; Vallacher and Nowak, 1997; Dale et al., 2014; Richardson et al., 2014). At this level of analysis, a more globally meaningful and perhaps, lawful description and understanding of social cognition is given through the emergent self-organizational patterns evident across the multiple scales comprising social units (e.g., Good, 2007; Coey et al., 2012). But to the best of our knowledge, no guiding framework has been provided to map the complexities and dynamics of social cognition across these levels of analysis. As an initial attempt towards this, we propose the schematic conveyed in Figure 3, which is our dynamical re-envisioning of Bohl and van den Bos’ (2012) framework.

A thorough review of dynamical systems as applied to social cognition and interaction is beyond the scope of the current paper, and has been elaborated elsewhere (Coey et al., 2012; Dale et al., 2014; Richardson et al., 2014). However, central to this, is that dynamical systems are typically characterized by coupling across different levels of organization (Wallot and Van Orden, 2012). The re-envisioning shown in Figure 3 is helpful because it highlights the different levels of organization inherent to the study of social cognition and coupling across the regions of the nervous system associated with Type 1 or Type 2 processes, personal-level experiences (P), bodies (B), and the (E) environment. This figure highlights the fundamentally embodied and embedded nature of agents engaging in social interaction. Therefore, while Figure 2 is a useful theoretical and analytical scaffold for mapping the relationship between social cues and signals, Figure 3 can be used as a means to map the interaction of the various components comprising social units that are coupled at a given point in an interaction, over various temporal scales (cf. Eiler et al., 2013). This is where we feel the methods of dynamical systems are useful as an integrative means to provide for the examination of social cognitive processes and refer the reader to Richardson et al. (2014) for an overview of how dynamical methods could be applied to map these relationships.

## Discussion

We have attempted to provide a framework for social cognition and perception in this paper with an explicit focus on the social information available in the environment. The next step is to map out how this work can generate a research program. Wilson and Golonka’s (2013) list of four questions to address for research examining embodied cognition serves as guidance for such a program. We briefly discuss these here. It is also important to point out that aspects of our framework complement what others have suggested with regard to additional social cognitive processes. For example, radical embodiment and dynamic coupling have been used to explain relational aspects of social cognition such as Communal Sharing relations and Authority Ranking relations (e.g., IJzerman and Koole, 2011; Beckes et al., 2014). However, for the present purposes we focus on the utilization of social information to understand and interact with others.

### What is the task to be solved?

Wilson and Golonka (2013) argue for performing a task analysis to better understand the nature of the behavior under investigation. From this, researchers should attempt to categorize and analyze social interactions by asking questions such as: What types of social interactions are there? What are the goals of the participants in such interactions? What kind of information is needed to achieve these goals, and how is it to be used to those ends? What is social cognition for in different types of interaction? One example is bargaining. In the simplest case there are two participants: a buyer and a seller. The goal of the buyer is to purchase a desired item at the lowest possible cost. The goal of the seller is to maximize profit by selling the item for the highest possible price. The act of bargaining is a social interaction that, when successful, solves the problem of the differences in these goals by settling on some value between the upper and lower bounds of desired prices. The buyer has in mind a price she is willing to pay, and the seller has in mind a price she is willing to sell for, and each participant attempts to determine what those values are in the mind of the other. Information about these values may be revealed during the course of negotiations. In a broad sense, across a variety of interactions, the task is to perceive the mind and intentions of others, but also to determine what actions and social goals others afford us.

### What resources are available to a participant in social interaction for solving this task?

At the basic level, as emphasized by Wilson and Golonka (2013), these resources are a body, including a nervous system, and an environment. The nervous system equips one with a perceptual apparatus. The body enables interaction. The environment contains the structure necessary to specify social information. Our integration of Gibsonian direct perception, the KSD principle, social cues and signals, situational semantics, projectable and non-projectable properties, and the Lens Model, provides a framework for determining an index of direct social perception and guiding the study of dynamic coupling across levels of social units. The broad goal is to show that the information to support social interaction exists, in principle. Kinematic analysis of the movements of others, Lens Model analysis (Cooksey, 1996; Hirschmüller et al., 2013) to examine the social cue and signal relationships, and geometric analysis (e.g., Nakayama and Loomis, 1974; Lee, 1976) of the information available to the perceptual systems, are required to answer this question. Importantly, one might consider how to determine whether a social cue, or set of social cues, veridically specifies a social signal. We have discussed in other work the degree to which techniques such as self-report, wisdom of the crowd, and expert ratings could be used to determine this (Wiltshire et al., 2014b). However, to some degree this is not necessary, though it would be quite informative. In other words, we do not assume that direct perception, or social perception more generally, must be accurate. Therefore, the approaches mentioned here for examining social cue and signal relationships are relative to the perceiver and evidence of direct perception of a social signal (perhaps, an inaccurate one) could be identified regardless.

### How are these resources assembled to solve the task?

Our approach suggests the possibility of a direct approach to social perception where perceptions are coupled with actions. Social interactions are perceptually guided, while at the same time generating new perceptual information. Research in social neuroscience, such as the study of M-pathways or motor resonance systems, alongside advances in the theory of intentional dynamics, can support this line of research. Following from the first question that there is a need to categorize social situations, there is a further need to categorize specific actions and interactions within these social situations. Defining the social action categories is a necessary step toward understanding how such actions are perceptually guided and organized, and further, how they become structured by intentional dynamics and in turn provide social information to the other. This aspect of a developing research program is captured in Proposition 1 and Proposition 2, above. The potential for lawful mapping between social cues and social signals may be dependent upon the situational characteristics of a given interaction. That is, certain situations may produce a lawful mapping between a set of social cues and a social signal, which can be perceived and processed automatically, quickly, and intuitively (see Wiltshire et al., 2014b for specific situational characteristics hypothesized to elicit such perceptions). It is by understanding not just the content of the social cues, but the context of the situation, that would facilitate understanding how social signals emerge from sets of social cues. Further, Proposition 6 makes an explicit claim about the relationship of a signal’s constituent social cues to one another. Together, these propositions provide for explicit testable hypotheses—and in the case of Proposition 6, is itself, a testable hypothesis—to examine how our framework of social cognition works to answer this question.

### During social interactions, do people perceive and use this information to guide interaction?

The answer to this question ultimately remains an empirical one for future work to demonstrate. Wilson and Golonka (2013) note that the typical procedure in perceptual research is the perturbation experiment. Once the perceptual information and relevant action have been identified, researchers should perturb the available information and observe the systematic effect this has on social interaction. In traditional perceptual research, perturbations are somewhat easy to achieve through the use of computer displays. However, the study of social interaction provides additional challenges. We have argued that the passive viewing of others in photographs or videos provides only a limited way to assess social perception, which is necessarily active and follows from social situations in which the observer is a participant and has goals to be achieved. Hence, there is a necessity of taking a perception-action approach to social perception. The use of virtual environments may provide a useful tool for engaging participants in social interactions while enabling experimenters to manipulate and perturb the available information. Our above Propositions 3, 4, and 5 touch on various aspects of this question. Propositions 3 and 4 make claims about the projectable nature of social cues and some social signals, and the non-projectable nature of other social signals. These propositions help lay the foundation for testing the perceivable nature of social cues and social signals. Proposition 5 takes an empirical position on the role of the nervous system, in particular the dorsal visual stream, in processing the action opportunities afforded by the directly perceived projectable properties. Lastly, determining whether someone perceived and actually uses social cues could be assessed using the cue utilization coefficients, which can be derived by having participants weight the cues after the fact or other physiological measures such as eye-tracking (Wiltshire et al., 2014b).

In conclusion, though traditional approaches to social cognition have produced intriguing explanations for how the brain processes social information, embodied and enactive approaches have shifted the consensus on the role of mental representations. Accordingly, understanding social cognition necessitates understanding an organism as embodied, situated, and acting within a data-rich social environment (e.g., Marsh et al., 2009a,b; Semin and Smith, 2013). In this article, we have built upon a framework meant to reconcile these approaches by relying on dual-process theories of cognition. We argued that this account was limited in its articulation of the prospects of direct social perception. The research propositions included throughout should serve to advance the science of social cognition through more systematic and theory-driven empirical examination. In this, we offer a path toward the reconciliation of radical and traditional approaches to social cognition. Our hope is that this effort contributes significantly towards understanding the complexity of what is perceived as the most socially intelligent creature known to walk the earth.

## Conflict of interest statement

The authors declare that the research was conducted in the absence of any commercial or financial relationships that could be construed as a potential conflict of interest.

## Footnotes

^1^Weaker versions of TT and ST also do not strictly assume that all mental states are unobservable; see Bohl and Gangopadhyay (2013).

^2^However, see Cook et al. (2014) for an alternative interpretation of mirror neuron function.

^3^To be clear, non-projectable properties are not to be confused with Withagen’s (2004) notion of nonspecifying variables, which are projectable, but do not contain specifying information.
